# Comparison of mechanical circulatory support with venoarterial extracorporeal membrane oxygenation or Impella for patients with cardiogenic shock: a propensity-matched analysis

**DOI:** 10.1007/s00392-020-01777-9

**Published:** 2020-11-13

**Authors:** Konstantinos Karatolios, Georgios Chatzis, Birgit Markus, Ulrich Luesebrink, Holger Ahrens, Dimitar Divchev, Styliani Syntila, Andreas Jerrentrup, Bernhard Schieffer

**Affiliations:** 1 Department of Cardiology, Angiology and Intensive Care, Philipps University Marburg, BaldingerStr., 35043 Marburg, Germany; 2 Department of Emergency Medicine, Philipps University Marburg, Marburg, Germany

**Keywords:** Mechanical circulatory support, Venoarterial extracorporeal membrane oxygenation, Impella, Cardiogenic shock

## Abstract

**Background:**

Percutaneous mechanical circulatory devices are increasingly used in patients with cardiogenic shock (CS). As evidence from randomized studies comparing these devices are lacking, optimal choice of the device type is unclear. Here we aim to compare outcomes of patients with CS supported with either Impella or vaECMO.

**Methods:**

Retrospective single-center analysis of patients with CS, from September 2014 to September 2019. Patients were assisted with either Impella 2.5/CP or vaECMO. Patients supported ultimately with both devices were analyzed according to the first device implanted. Primary outcomes were hospital and 6-month survival. Secondary endpoints were complications. Survival outcomes were compared using propensity-matched analysis to account for differences in baseline characteristics between both groups.

**Results:**

A total of 423 patients were included (Impella, *n* = 300 and vaECMO, *n* = 123). Survival rates were similar in both groups (hospital survival: Impella 47.7% and vaECMO 37.3%, *p* = 0.07; 6-month survival Impella 45.7% and vaECMO 35.8%, *p* = 0.07). There was no significant difference in survival rates, even after adjustment for baseline differences (hospital survival: Impella 50.6% and vaECMO 38.6%, *p* = 0.16; 6-month survival Impella 45.8% and vaECMO 38.6%, *p* = 0.43). Access-site bleeding and leg ischemia occurred more frequently in patients with vaECMO (17% versus 7.3%, *p* = 0.004; 17% versus 7.7%, *p* = 0.008).

**Conclusions:**

In this retrospective analysis of patients with CS, treatment with Impella 2.5/CP or vaECMO was associated with similar hospital and 6-month survival rates. Device-related access-site vascular complications occurred more frequently in the vaECMO group. A randomized trial is warranted to examine the effects of these devices on outcomes and to determine the optimal device choice in patients with CS.

## Introduction

Despite the increased use of evidence-based medicine and interventions, CS still portends unacceptably high hospital mortality rates. Percutaneous mechanical circulatory support (MCS) devices are increasingly used in patients with CS, in order to restore hemodynamics, improve cardiac output and ensure adequate end-organ perfusion [1]. The Impella pump and venoarterial extracorporeal membrane oxygenation (vaECMO) are the most frequently used devices for temporary percutaneous MCS in this context. However, the physiologic effects on cardiac work and hemodynamics, insertion technique as well as clinical management differ significantly between these two devices [2, 3]. Despite the growth in the use of vaECMO and Impella to support patients with CS, no randomized trials have compared the efficacy of these devices in patients with CS. As a result, optimal selection of the device type is still a matter of debate and no specific guideline recommendation exists. Therefore, the aim of this retrospective study was to compare outcomes of patients with CS supported with either Impella (2.5/CP) or vaECMO.

## Methods

### Study design

We retrospectively analyzed data from all consecutive patients supported with Impella 2.5/CP or vaECMO for CS in our institution, a European tertiary University Hospital, from September 2014 to September 2019. Patients with refractory cardiac arrest in whom insertion of the device took place under ongoing cardiopulmonary resuscitation were excluded from the analysis. CS was defined as the need for continuous infusion of catecholamines to maintain systolic blood pressure > 90 mmHg with clinical signs of pulmonary congestion and signs of end-organ hypoperfusion indicated by an elevated lactate level > 2  mmol/L. Operators used MCS in patients with CS, according to our institutional practices. However, as there is insufficient evidence for the choice of the circulatory support device type in CS and neither the superiority of one device over the other was proven in the setting of CS, the decision to implant an Impella or vaECMO was based on operator’s clinical evaluation (which was based on individual patients’ parameters, including severity of shock, prior resuscitation, bleeding risk, isolated left ventricular failure, biventricular failure, combined cardiorespiratory failure). In brief, the Impella device is our first choice of MCS in patients with CS due to isolated left ventricular (LV) failure. In cases with biventricular failure or cases with combined LV and pulmonary failure we implant a vaECMO first. If vaECMO leads to LV distension and worsening pulmonary edema, an Impella device for venting is additionally inserted. The development of respiratory failure, right heart failure, hemodynamic deterioration, progressive multiorgan failure while on Impella support is, according to our institutional algorithm, an indication for additional implantation of vaECMO. Besides mechanical circulatory support, all patients received standardized medical care and management in accordance with guidelines. If patients received ultimately both devices, they were analyzed according to the device first implanted.

The investigation conforms with the principles outlined in the Declaration of Helsinki. The study was approved by the local ethics committee of the Philipps University of Marburg. The need for informed consent was waived due to the retrospective nature of the study.

### Patients’ management

All MCS devices were implanted percutaneously in the catheterization laboratory by experienced operators. The Impella pump (Abiomed) was implanted through the femoral artery and placed via the retrograde approach through the aortic valve into the left ventricle under fluoroscopic control. The vaECMO (Maquet Getinge Group) was inserted percutaneously using arterial (17F) and venous (21F for female and 23F for male) femoral cannulas with an additional antegrade femoral limb perfusion cannula. All cardiac arrest patients were treated with targeted temperature management (mild hypothermia of 34 °C) for 24 h with an endovascular cooling device (Thermogard Temperature Management System, Zoll). Inotropes and vasopressors were used to obtain a mean arterial pressure ≥ 65 mmHg. Circulatory support flow was adjusted to maintain mean arterial pressure ≥ 65 mmHg with the lowest possible dose of catecholamines and to cover metabolic needs as assessed by central venous oxygen saturation (≥ 70%) and serum lactate levels (< 2.0 mmol/L). The decision to wean the circulatory support device was based on resolution of shock and clinical assessment. Weaning process was performed by gradually decreasing support. Once the support of the device was reduced to low levels (for Impella performance level 1 and for vaECMO < 1.5 L/min) with stable mean arterial pressure ≥65 mmHg, no or low doses of catecholamines, central venous oxygen saturation ≥7 0% and serum lactate levels < 2.0 mmol/L the device was removed in ICU and hemostasis was achieved with mechanical compression (St. Jude Medical FemoStop).

### Data collection and outcome variables

Intrahospital data, outcomes and follow-up data were collected from the medical charts. Our primary outcomes were survival to hospital discharge and at 6-month. Secondary endpoints were complications. Complications included device-related vascular complications (bleeding at access-site requiring transfusion, limb ischemia requiring removal of the device, surgery or interventional repair), myocardial reinfarction, stroke and other non-device-related bleeding. Access-site bleeding requiring transfusion was defined as bleeding at the cannulation site with need of transfusion of at least two red blood cell (RBC) units. Cerebral functional status in cardiac arrest patients was determined according to the Pittsburgh cerebral performance category (CPC) based on medical records or discharge summary abstracts.

### Statistical analysis

All data were analyzed retrospectively. Data are presented as absolute variables and percentages (%) for categorical variables and either median with interquartile range [IQR 25th–75th percentile] or mean with standard deviation according to the distribution of the variables. We assessed normality using Shapiro–Wilk test as well as Pearson Tests. After testing for normal distribution, Student’s t test or Mann–Whitney test was implemented to test for differences between the various characteristics. For categorical variables Fisher’s exact test or Chi-square test were used, as appropriate. Interactions between nominal variables were measured with lambda coefficient. Patients at risk were assessed with the log-rank test of the survival analysis. The variables were dichotomized according to median in overall population, when they were not linearly distributed. An initial analysis was performed in order to identify the variables associated with outcome mortality in the overall population. A separate analysis was performed in order to identify the variables with a different distribution among the groups of devices. All these variables are presented in the Table 1. Age, Charlson Comorbidity Index (CCI), vasoactive score, creatinine, GFR, pH, PaO_2_/FiO_2,_ etiology of cardiogenic shock and prior CPR were included in the model as significantly associated with outcome in univariate analysis or as clinically meaningful. Propensity score matching was used to balance observed covariates in treatment groups [4, 5]. In this study, the propensity score was the conditional probability for getting vaECMO for CS, as a binary dependent variable, under a set of measurements. Age, CCI, vasoactive score, creatinine, pH, etiology of shock, PaO_2_/FiO_2_ and prior CPR were added into a multivariable logistic regression model. The predicted probability derived from the logistic equation was used as the propensity score for each individual. The model revealed a c-statistic of 0.88 (C.I. 0.81–0.95). Then we performed a conditional logistic regression after matching on the propensity score in a 1:1 in order to identify the matched pairs. Given the frequency of the outcome in total population, our power calculation showed that we would need a minimum of 75 pairs in order to establish our superiority or non-inferiority hypothesis with a non-inferiority or superiority margin of − 0.1 with a power of 0.8 and type α error of 0.05. All analyses were considered statistically significant for *p* < 0.05. All analyses were two-sided. Statistical analysis was performed using SPSS 24 and Graphpad Prim 6.0.

**Table 1:** Demographics and baseline characteristics

Variable	Overall cohort			Matched cohort		
	Impella (*n* = 300)	vaECMO (*n* = 123)	*p* value	Impella (*n* = 83)	vaECMO (*n* = 83)	*p* value
Age (years)	68.96 ± 11.56	61.25 ± 10.40	< 0.001	63.71 ± 11.91	62.82 ± 10.73	0.61
Male, *n* (%)	229 (76.3)	101 (78.2)	0.24	65 (78.3)	66 (79.5)	1
BMI (kg/m^2^)	28.56 ± 5.01	28.42 ± 3.57	0.79	29.71 ± 5.3	28.84 ± 3.5	0.21
Baseline LVEF (%)	35.55 ± 4.26	34.7 ± 4.53	0.07	34.88 ± 4.55	34.45 ± 4.8	0.56
Etiology of cardiogenic shock			0.23			1
DCM/myocarditis	38 (12.6)	21 (17)		12 (14.4)	12 (14.4)	
AMI	262 (87.4)	106 (83)		71 (85.6)	71 (85.6)	
Impella						
2.5 CP	227 (75.7) 73 (24.3)	–		66 (79.5) 17 (20.5)	–	
STEMI on presentation, *n* (%)	139 (46.3)	60 (48.7)	0.67	34 (40.9)	38 (45.8)	0.64
Mechanical ventilation on admission, *n* (%)	236 (78.7)	101 (82.1)	0.2	65 (78.3)	65 (78.3)	1
Prior cardiac arrest, *n* (%)	117 (39)	72 (58.1)	0.0004	38 (45.7)	40 (48.2)	0.88
OHCA, *n* (%)	77 (25.7)	50 (40.6)		29 (34.9)	30 (36.1)	
IHCA, *n* (%)	40 (35.3)	22 (17.9)		9 (10.8)	10 (12)	
Medical comorbidities						
Hypertension, *n* (%)	192 (64)	85 (69.1)	0.83	50 (60.2)	62 (74.5)	0.07
Diabetes, *n* (%)	85 (28.3)	41 (33.3)	0.35	29 (35)	31 (37.3)	0.31
PAD, *n* (%)	58 (19.3)	21 (17)	0.42	16 (19.3)	11 (13.3)	0.4
Prior CAD, *n* (%)	129 (43)	47 (38.2)	0.39	40 (48.2)	33 (40)	0.35
Stroke, *n* (%)	32 (10.7)	19 (15.4)	0.19	14 (16.9)	11 (13.3)	0.67
Prior PCI, *n* (%)	93 (31)	34 (27.6)	0.56	28 (33.7)	26 (31.3)	0.87
CABG, *n* (%)	35 (11.7)	13 (10.6)	0.87	10 (12)	9 (10.8)	1
Prior HF, *n* (%)	42 (14)	16 (13)	0.88	10 (12)	12 (14.5)	0.82
Prior MI, *n* (%)	70 (23.3)	27 (22)	0.8	24 (28.9)	23 (27.7)	1
Charlson comorbidity index	4.66 ± 2.32	3.98 ± 2.4	0.007	4 [3, 5]	4 [3, 5]	1
Catecholamines						
Vasopressors or inotropes, *n* (%)	300, (100)	123, (100)	1	83 (100)	83 (100)	1
Vasoactive Score	51.15 [18.89, 86.9]	54.51 [30, 81.22]	0.38	49.28 [19.75, 87.16]	51 [30, 78]	0.64
Hemodynamic variables on admission						
Heart rate (bpm)	94 ± 20.6	94.6 ± 19.67	0.78	98.16 ± 20.71	95.88 ± 22.49	0.5
Systolic blood pressure (mmHg)	106.1 ± 30.93	101.5 ± 22.67	.014	108 ± 35.18	101.3 ± 24.53	0.06
Diastolic blood pressure (mmHg)	64.13 ± 15.73	61.34 ± 12.55	0.08	64.71 ± 17.39	60.93 ± 12.09	0.11
Mean blood pressure (mmHg)	78.1 ± 19.6	74.71 ± 14.44	0.08	80.04 ± 22.11	74.37 ± 14.91	0.06
Blood values on admission						
Lactate (mmol/l)	6.5 ± 4.7	9.18 ± 5.63	<0.001	6.82 ± 5.04	7.97 ± 4.8	0.14
GFR (ml/min)	51 [38.48, 63.75]	47 [33, 63]	0.11	51 [38, 68]	48.5 [33.5, 63]	0.23
Arterial pH	7.27 ± 0.17	7.21 ± 0.19	0.001	7.25 ± 0.19	7.26 ± 0.18	0.84
PaO_2_/FiO_2_	232 [175, 327]	226 [155, 306]	0.23	233 [172, 306]	222 [147, 273]	0.31
Creatinine (mg/dl)	1.51 [1, 1.73]	1.47 [1.3, 1.94]	0.55	1.45 [1.1, 1.68]	1.4 [1.1, 1.85]	0.82
Hemoglobin (g/dl)	126 [120, 129]	123 [113, 140]	0.1	123 [116, 139]	123 [115, 140]	0.74
Sodium (mmol/l)	137 ± 4.2	137 ± 4.51	0.23	136.6 ± 5.17	137 ± 4.8	0.47
Potassium (mmol/l)	4.2 [3.9, 4.3]	4.1 [3.9, 4.4]	0.08	4.2 [3.9, 4.23]	4.1 [3.9, 4.4]	0.63
Thrombocytes (G/l)	219 [190, 262]	234 [157, 709]	0.45	217 [172, 262]	240 [158, 709]	0.81

*BMI* body mass index, *LVEF* left ventricular ejection fraction, *DCM* dilated cardiomyopathy, *AMI* acute myocardial infarction, *STEMI* ST elevation myocardial infarction, *OHCA* out-of-hospital cardiac arrest, *IHCA*, intrahospital cardiac arrest, *PAD* peripheral artery disease, *CAD* coronary artery disease, *PCI* percutaneous coronary intervention, *CABG* coronary artery bypass-graft, *HF* heart failure, *MI* myocardial infarction, *GFR* glomerular filtration rate; Numbers are presented as mean (± standard deviation), median [interquartile range, IQR 25th–75th percentile] or frequency (percentile)

## Results

### Patients

From September 2014 to September 2019, a total of 423 patients with CS were treated with MCS. Among them, 300 patients (71%) underwent Impella implantation and 123 patients (29%) were supported with vaECMO. Within the Impella group, 227 patients (75.7%) were supported with an Impella 2.5 and 73 (24.3%) patients with Impella CP. Forty-two (9.9%) patients were ultimately supported by both devices simultaneously (20 patients Impella first and 22 patients vaECMO first).

Demographic and baseline characteristics of patients are presented in Table 1. On admission, the groups were similar regarding gender distribution, cardiovascular risk factors, etiology of CS, mean blood pressure, LVEF, inotropic/vasopressor therapy and creatinine values. Compared to patients in the Impella group, patients in the vaECMO group were younger (68.96 ± 11.56 versus 61.25 ± 10.4, *p* < 0.001) and presented more often with cardiac arrest (58.9% versus 39%, *p* = 0.0004) (Table 1). Also, vaECMO supported patients had significantly worse pH (7.27 ± 0.17 versus 7.21 ± 0.19, *p* = 0.001) and significantly higher lactate levels (6.5 ± 4.7 versus 9.18 ± 5.63 mmol/l, *p* < 0.001). Using propensity score, 83 pairs of patients were matched. The characteristics of the propensity-matched cohort were well balanced and evenly distributed regarding covariates (Table 1). The procedural characteristics of the vaECMO and Impella supported patients (overall and matched cohort) are displayed in Table 2.

**Table 2:** Procedural characteristics of the overall and matched cohort

Variable	Overall cohort			Matched cohort		
	Impella (*n* = 300)	vaECMO (*n* = 123)	*p* value	Impella (*n* = 83)	vaECMO (*n* = 83)	*p* value
Door to MCS (min)	356.6 ± 87.81	350.4 ± 84.21	0.76	353.3 ± 84.18	342.6 ± 85.9	0.42
Duration of support (hours)	128.3 ± 37.21	128.3 ± 43.78	0.04	118.8 ± 36	130.2 ± 47.2	0.76
Culprit vessel, *n* (%)^a^			NS			NS
Left main	33 (12.6)	6 (5.7)		11 (10)	12 (16.9)	
LAD	125 (47.7)	47 (44.3)		28 (39.4)	28 (39.4)	
LCx	44 (16.8)	19 (17.9)		16 (22.5)	16 (22.5)	
RCA	48 (18.3)	25 (23.5)		10 (13.7)	10 (92.5)	
Bypass-graft	12 (4.5)	9 (8.5)		4 (15, 4)	5 (7.5)	
Multivessel disease	141 (53.8)	74 (69.8)	0.0051	42 (66.7)	42 (64.6)	1
Multivessel Intervention	89 (34)	39 (36.7)	0.63	28 (38.3)	24 (33.8)	0.6
Unsuccessful PCI	13 (4.9)	3 (2.9)	0.57	2 (2.8)	0 (0)	0.5
Contrast Agent (ml)	255 [180, 320]	250 [180, 270]	0.06	255 [200, 350]	250 [180, 270]	0.004

*MCS* mechanical circulatory support, *LAD* left coronary artery, *LCx* left circumflex artery, *RCA* right coronary artery, *PCI* percutaneous coronary intervention. Numbers are presented as mean (± standard deviation), median [interquartile range, IQR 25th–75th percentile] or frequency (percentile); *NS* non-significant

^a^Percentile refers to the patients with AMI

### Outcome

**Fig. 1:**
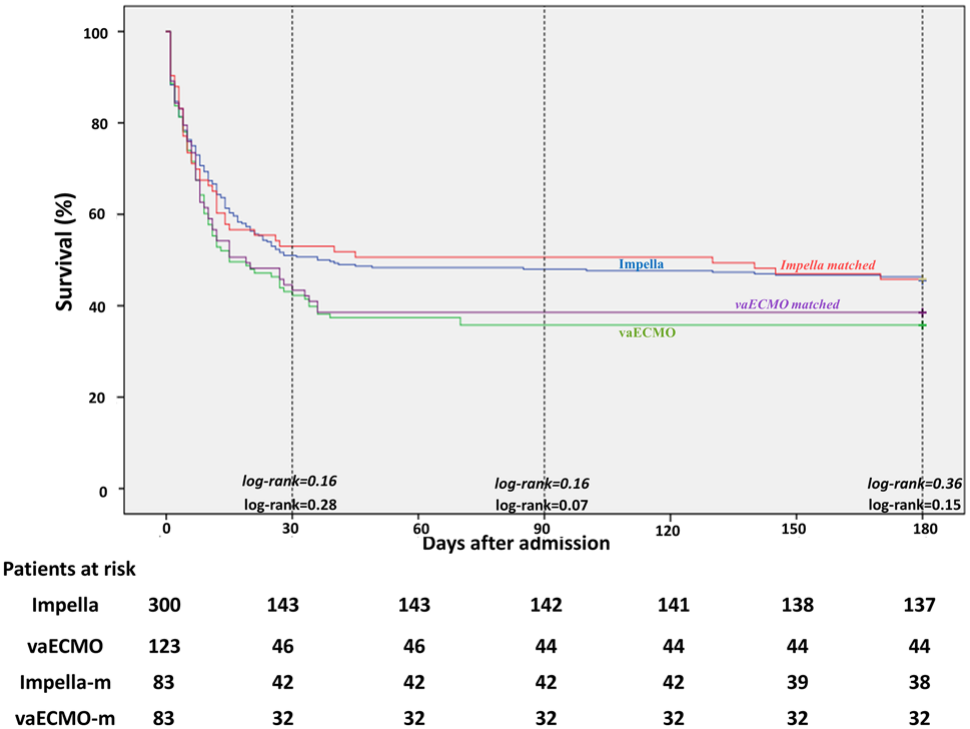
Kaplan–Meier curves demonstrating 6-month survival in Impella and vaECMO patients. *Impella-m* Impella-matched cohort, *vaECMO-m* vaECMO-matched cohort

**Fig. 2:**
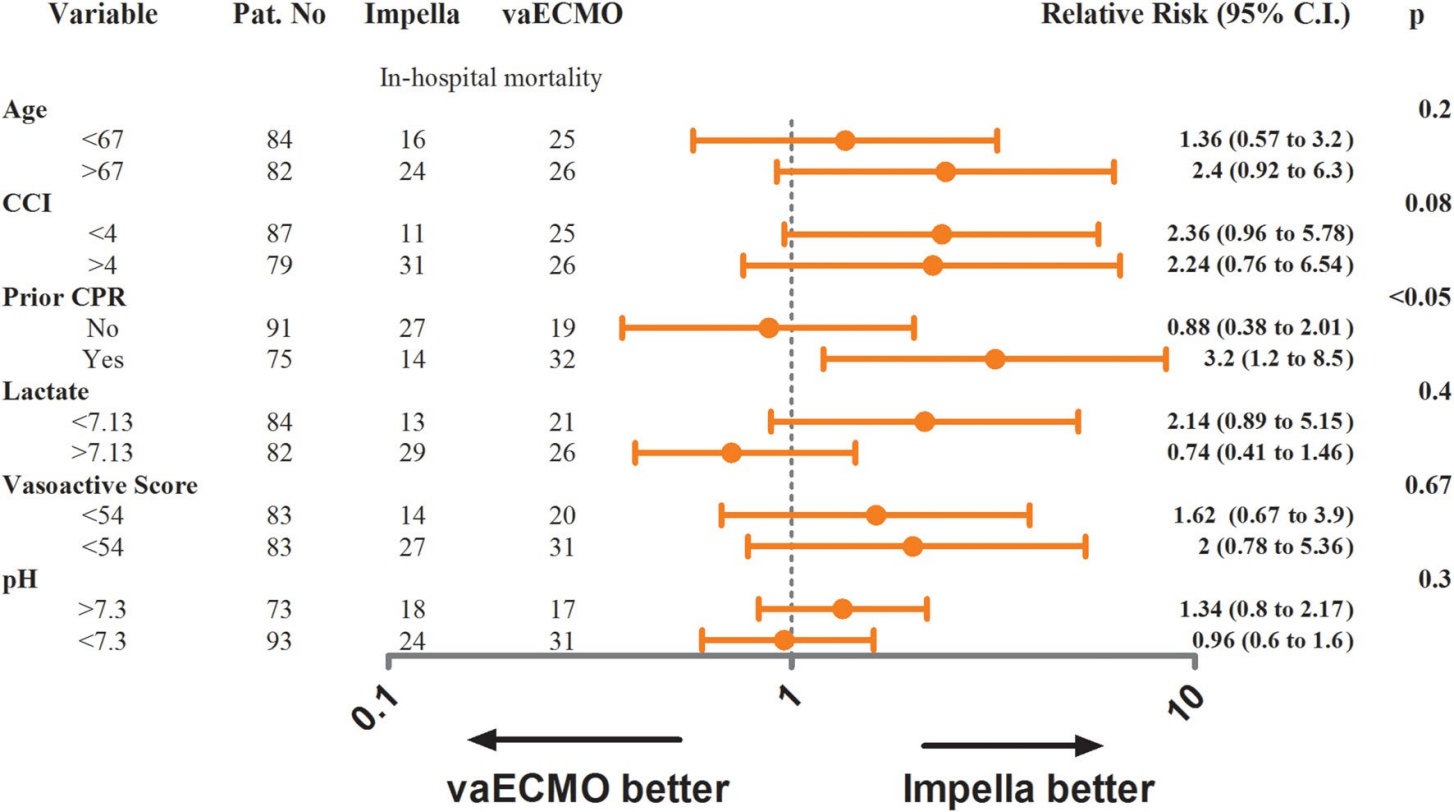
Forest plot displaying the relative risk of different tested subgroups for the primary endpoint hospital mortality in matched cohort

Information on hospital and 6-month mortality were available for all patients. Hospital survival rates were 47.7% in the Impella group and 37.3% in the vaECMO group (*p* = 0.07) (Table 3). Six-month survival rates were 45.7% and 35.8% (*p* = 0.08) for the Impella and vaECMO group, respectively. In the matched cohort, the hospital and 6-month survival rates were also comparable for the Impella and vaECMO group (hospital survival: 50.6% versus 38.6%, *p* = 0.16 and 6-month survival: 45.8% versus 38.6%, *p* = 0.43) (Fig. 1 and Table 3). Furthermore, survival rates were comparable across all tested subgroups except for patients with prior cardiac arrest where Impella support improved the primary endpoint of hospital survival (Fig. 2). Patients with pre-percutaneous coronary intervention (PCI) Impella (*n* = 94) had better survival rates than patients with post-PCI Impella (*n* = 151) implantation (hospital survival and 6-month survival: 63.8% versus 51%, *p* = 0.03).

**Table 3:** Outcomes

Outcome	Impella (*n* = 300)	vaECMO (*n* = 123)	*p* value	Impella-matched (*n* = 83)	vaECMO-matched (*n* = 83)	*p* value
Survival to hospital discharge, *n* (%)	143 (47.7)	46 (37.3)	0.07	42 (50.6)	32 (38.6)	0.16
Survival at 6-month, *n* (%)	137 (45.7)	44 (35.8)	0.07	38 (45.8)	32 (38.6)	0.43
Access-site bleeding requiring transfusion, *n* (%)	22 (7.3)	21 (17)	0.004	10 (12)	12 (9.8)	0.82
Limb ischemia requiring intervention, *n* (%)	23 (7.7)	21 (17)	0.008	7 (8.4)	12 (14.5)	0.33
Myocardial Reinfarction, *n* (%)	4 (1.3)	2 (1.6)	1	1 (1.2)	1 (1.2)	1
Stroke, *n* (%)	5 (1.7)	3 (2.4)	0.7	2 (2.4)	2 (2.4)	1
Non-device-related bleeding, *n* (%)	6 (2)	9 (7.3)	0.02	2 (2.4)	7 (8.4)	0.17

Numbers are presented as mean (± standard deviation), median [interquartile range, IQR 25th–75th percentile] or frequency (percentile)

Regarding complications, similar rates of myocardial reinfarction (Impella: 1.3% versus vaECMO: 1.6%, *p* = 0.1) and stroke (Impella: 1.7% versus vaECMO: 2.4%, *p* = 0.7) were observed (Table 3). Device-related access-site vascular complications occurred more frequently in the vaECMO group than in the Impella group. Access-site bleedings rates requiring transfusion were significantly higher within the vaECMO group (17% versus 7.3%, *p* = 0.004). Vascular access-site ischemic complications requiring removal of the device, surgery or percutaneous intervention occurred in 17% and 7.7% (*p* = 0.008) of patients in the vaECMO and Impella group, respectively (Table 3).

## Discussion

We here describe a large single-center, propensity-matched study of patients with CS supported with either Impella 2.5/CP or vaECMO. To our knowledge, this is the largest single-center cohort comparing Impella and vaECMO in patients with CS. In our analysis, Impella 2.5/CP and vaECMO were associated with similar outcome in patients with CS. In the overall cohort, hospital and 6-month survival rates were comparable among the Impella and vaECMO group. However, several baseline characteristics lead to consider the vaECMO group a higher-risk population over the Impella group. Patients of the vaECMO group had a higher percentage of prior cardiac arrest, higher baseline lactate levels and lower pH. These factors are known to be associated with worse outcome in CS patients [6–8]. Therefore, since we documented significant differences in baseline characteristics especially with respect to critical prognostic parameters we performed a propensity-matching to enhance comparability between the two groups. After propensity-matching, the baseline characteristics of the matched cohort were comparable and evenly distributed between the Impella and vaECMO group. After propensity-matching, Impella and vaECMO supported patients showed again comparable hospital and 6-month survival rates. This result was consistent among all tested subgroups, in contrast to patients with prior cardiac arrest in whom Impella support was associated with improved hospital survival rates (Fig. 2). In the particular group of patients with post-cardiac arrest shock and vaECMO support high rates of access-site complications (up to 29%) were previously reported [9, 10]. In accordance, patients in the vaECMO group with prior cardiac arrest had, in our analysis, device-associated vascular complications in 23% of cases (versus 8.4% in Impella patients with prior cardiac arrest), which may have contributed to the result in this subgroup. However, the subgroup analysis of cardiac arrest patients should be interpreted with caution and needs further evaluation in a sufficiently powered study.

Given the persistently poor outcomes in patients with CS, interest in the role and appropriate selection of these two invasive devices has been increasing. However, the selection of type of MCS in patients with CS is still unclear and varies among institutions, since solid evidence from randomized controlled trial are, so far, lacking. Therefore, comparative analyses of real-life use of MCS in patients with CS regarding outcomes and complications as presented in our investigation are important and add information to the current knowledge. To date, very few investigations compared outcomes of patients treated with these two MCS devices [11–14] and no randomized data are available favoring one type of device over the other. Recently, Garan and colleagues, prospectively compared outcomes of Impella and vaECMO in 51 patients with acute myocardial infarction (AMI)-related CS [13]. In accordance with our results, their data demonstrated that the two devices were associated with similar outcomes [13]. Karami and colleagues conducted a retrospective study of CS patients requiring Impella or vaECMO [14]. In this study, Impella and vaECMO support was again not associated with a difference in 30-day mortality. Furthermore, in another retrospective analysis by Schiller and colleagues, short and long-term survival was not measurably different between Impella and vaECMO supported patients with CS, even after adjustment of disease severity through the SAVE score [11].

Randomized trials adequately powered are particularly challenging to conduct in patients with CS. Several randomized trials investigating MCS in CS, like the DanGer SHOCK (NCT01633502), ECLS-SHOCK (NCT03637205) and EUROSHOCK (NCT03813134) trial, are ongoing and their results awaited. However, these trials compare either vaECMO or Impella to standard medical treatment. The lack of an appreciably and clear benefit of one device over the other, warrants, in our view, also a randomized controlled trial comparing head-to-head these two devices in patients with CS.

Regarding safety outcomes, we found no differences in stroke or myocardial reinfarction between Impella and vaECMO supported patients. However, as expected access-site complications occurred more frequently in the vaECMO group than in the Impella group. This finding is in line with previous investigations [14–16]. Recently, a large-scale propensity-matched, registry-based retrospective cohort study observational including 1768 patients with CS treated with Impella reported high rates of major bleeding of 31.3% [17], which was markedly higher compared to the bleeding rates in our Impella cohort. On the other side, further retrospective studies reported lower vascular complication rates comparable to our results [15, 16]. Registry data including 112 patients supported with Impella for AMI-related CS reported an overall vascular complication rate of 17% [15]. Limb ischemia occurred in 3.5% and major access-site bleeding in 9.8% of patients [15]. Another retrospective analysis of 237 patients with AMI-associated CS treated with Impella reported peripheral ischemic vascular complications in 9.8% of Impella patients [16]. In patients treated with vaECMO vascular complications occur at a high rate as repeatedly reported in several investigations. A meta-analysis of 1866 patients supported with vaECMO for CS reported major bleeding rates of 40.8% and a lower extremity ischemia rate of 16.9% [18], whereas the study by Combes and colleagues reported femoral bleeding in 32% and peripheral leg ischemia in 20% of vaECMO patients [19]. The higher rates of peripheral vascular complications in the vaECMO group as compared to the Impella group might be attributed to the larger vascular access needed for vaECMO implantation.

## Limitations

Even though this might be the largest comparison of vaECMO versus Impella, this study has several limitations. First, our observations are obviously limited by the retrospective and non-randomized design of our study and therefore, prone to selection bias. Second, our investigation was a single-center study, but based on otherwise standardized procedures. Third, the device selection was based on operator’s discretion and our institutional algorithm and not directed by a study protocol.

But beside some limitations, the strength of this matched analysis is that it is, so far, the largest single-center investigation comparing CS patients treated with either Impella 2.5/CP or vaECMO.

Thus, since our results are preliminary, the association between these two MCS devices and outcomes of patients with CS warrants further evaluation in randomized studies.

## Conclusions

In this retrospective analysis of a large cohort of patients with CS, treatment with Impella 2.5/CP or vaECMO was associated with similar hospital and 6-month survival rates. Device-related access-site vascular complications occurred more frequently in patients with vaECMO support than in Impella patients. A randomized trial is warranted to examine the effects of these MCS devices on outcomes and to determine the optimal device choice in patients with CS.
